# The genetic and clinical characteristics of oculopharyngeal muscular dystrophy patients in Israel

**DOI:** 10.1186/s13023-026-04313-6

**Published:** 2026-05-19

**Authors:** Merav Ben-David, Lior Greenbaum, Vera Nikitn, Alex Zvulunov, Hagit Charas, Naama Divon, Tali Barkan, Odelia Chorin, Haike Reznik-Wolf, Ofira Zloto, Limor Benyamini, Shahar Shelly, Amir Dori

**Affiliations:** 1 Department of Neurology, Sheba Medical Center, Tel Hashomer, Ramat Gan, Israel; 2 The Danek Gertner Institute of Human Genetics, Sheba Medical Center, Tel Hashomer, Ramat-Gan, Israel; 3 Gray Faculty of Medical & Health Sciences, Tel Aviv University, Tel Aviv, Israel; 4 Multidisciplinary Service for OPMD Patients, Sheba Medical Center, Tel Hashomer, Ramat-Gan, Israel; 5 Recanati School of Medicine, Reichman University, Herzliya, Israel; 6 The Non-Profit Organization for Promotion of Health and Cure of OPMD, Bnei Dror, Israel; 7 The Genetics Institute and Genomics Center, Tel Aviv Sourasky Medical Center, Tel Aviv, Israel; 8 Department of Ophthalmology, Sheba Medical Center, Tel Hashomer, Ramat Gan, Israel; 9 Department of Otolaryngology, Head and Neck Surgery, Sheba Medical Center, Tel Hashomer, Ramat Gan, Israel; 10 Department of Neurology, Rambam Medical Center, Haifa, Israel; 11 Rappaport Faculty of Medicine, Technion, Haifa, Israel; 12 Department of Neurology, Mayo Clinic, Rochester, MN USA

**Keywords:** Oculopharyngeal muscular dystrophy, Myopathy, Trinucleotide repeat expansion, *PABPN1*

## Abstract

**Background:**

Oculopharyngeal muscular dystrophy (OPMD) is a late-onset autosomal dominant myopathy, caused by a (GCN)n/polyalanine repeat expansion in the *PABPN1* gene. In Israel, OPMD is particularly prevalent among individuals of Jewish Bukharian descent due to a (GCN)13 repeat expansion. In this retrospective study, we collected genetic and clinical data of OPMD patients who visited the Israeli reference clinic from its opening in 2022 through September 2025 and were enrolled in the Israeli OPMD registry (IsrO-PMD).

**Results:**

A total of 102 Jewish individuals with OPMD symptoms and a confirmed molecular diagnosis were identified (52 males, 51.0%). The heterozygous (GCN)13 repeat allele (10/13 genotype) was found in 95 patients (93.1%), of whom 89 (93.7%) were of Bukharian descent, five (5.6%) were of Bulgarian origin, and a single patient was from Georgia. In this 10/13 genotype group (*n* = 95), initial symptoms were dysphagia and ptosis, which occurred at a similar mean age of 53.0 ± 8.3 and 53.9 ± 7.2 years, respectively. Symptoms of limb muscle weakness occurred significantly later, at a mean age of 57.9 ± 10.2 years. Overall, the onset of symptoms occurred approximately 4 years earlier in females (48.9 ± 7.9, *n* = 48) than in males (52.6 ± 7.1, *n* = 47), and this difference was statistically significant (*p* = 0.02). However, the difference did not reach significance when calculated for each of the three symptoms separately. On evaluation, at a mean age of 61.6 ± 10.7 years, dysphagia was present in 92/95 (96.8%) of patients, ptosis in 85/95 (89.5%), and limb muscle weakness in 56/95 (58.9%) of cases, most notably of hip flexion and abduction. Different genotypes were detected in seven patients: Three of Bukharian descent were homozygous for (GCN)13 repeats, three of Karaite descent were homozygous for (GCN)11 repeats, and one of Tunisian origion was heterozygous for (GCN)15 repeats (10/15 genotype).

**Discussion:**

While most OPMD patients in Israel are of Jewish Bukharian descent, patients from other origins were also identified. Dysphagia and ptosis are the common initial symptoms, and overall, the onset of symptoms was reportedly earlier in females. Proximal muscle weakness becomes common with disease progression. Further studies are required to delineate genotype-phenotype correlations.

## Background

Oculopharyngeal muscular dystrophy (OPMD) is a rare, late-onset myopathy characterized by dysphagia and upper eyelid ptosis, typically appearing in the fifth or sixth decade of life [1, 2]. The reported mean age of symptoms’ onset is approximately 48 years for ptosis and 50 for dysphagia [3]. Proximal limb weakness may occur later, as well as other neurological manifestations; however, the rate of disease progression is generally slow [4–6].

The cause of OPMD (MIM #164,300) is an expansion of a trinucleotide (GCN) repeat in exon 1 of the polyadenylate binding protein nuclear 1 (*PABPN1*) gene, previously named *PABP2* [7, 8]. The wild-type allele contains up to 10 alanine repeats, while in OPMD, it expands to include 11–18 repeats [2, 9, 10], leading to elongation of the polyalanine N-terminal domain of the protein. OPMD is inherited in an autosomal dominant (AD) manner, whereas patients with biallelic repeat expansions have also been described, typically with a more severe disease course [3, 11]. Rarely, the polyalanine expansion is caused by a point mutation [12]. Among heterozygous patients, there is likely a correlation between expansion length and the age of symptom onset [2].

The *PABPN1* gene encodes a nuclear protein that binds to messenger RNA and activates poly(A) polymerase, thus regulating poly(A) tail polymerization and mRNA stability [13–15]. Muscle biopsy in OPMD patients demonstrates dystrophic changes, rimmed vacuoles, and intranuclear inclusions [16, 17]. Nevertheless, the *PABPN1* gene is ubiquitously expressed in body cells, and it is unclear why it primarily affects specific muscles [18].

OPMD is a rare disease worldwide, with an estimated prevalence of 1:100,000 in the European population [3, 19], but is disproportionately prevalent among several populations, including Bukharian Jews [20–23], French Canadians [24], Hispanic New Mexicans [25], and in the Canary Islands’ population [26]. The Bukharian Jewish population originated from the Jewish diaspora, settling in the geographical region that is today Uzbekistan. A cluster of OPMD patients within this ancestry was reported and described approximately 30 years ago by Blumen et al. with a minimal estimation of OPMD prevalence of 1:600 in this population [23]. Among Bukhara Jews, OPMD is caused by an expanded (GCN)13 allele, attributed to a founder effect that occurred ~800 years ago in a population living in relatively closed communities with a high rate of consanguinity [20–22].

Recently, a national Israel registry for OPMD (IsrO-PMD) was established, aiming to study the clinical features and natural history of OPMD and to facilitate research toward the development of therapeutic agents. The rationale and design of the registry were detailed by Stein et al. [27]. Here, we describe the clinical manifestations of 102 genetically characterized OPMD patients recruited during the first 4 years of the IsrO-PMD registry, all of whom were evaluated in a single neuromuscular clinic.

## Methods

### Study design

This is an observational retrospective study based on the Sheba Medical Center (SMC) IsrO-PMD cohort database. Inclusion criteria were (1) symptoms and signs consistent with OPMD; (2) Genetic diagnosis of OPMD based on testing of (GCN)n allele expansion in *PABPN1*; (3) written informed consent to participate in the study.

All participants were evaluated at the OPMD clinic of the SMC neuromuscular unit, a tertiary referral center in Israel, from its establishment in 2022 to September 2025. This clinic was recognized in 2023 by the Israeli Ministry of Health as a national specialized clinic for OPMD. This is a multidisciplinary service for OPMD patients and includes specialists in neurology, ear, nose, and throat (ENT) and speech therapy, and ophthalmology. As such, patients were referred from all geographic regions of Israel and by all health maintenance organizations (HMOs). The SMC Institutional Review Board approved the study (8801–21-SMC).

### Clinical evaluation

Demographic data, family history details, and genetic testing results were systematically recorded. Clinical evaluation was performed by a board-certified neurologist specializing in neuromuscular disorders. This included a medical history review focusing on signs and symptoms relevant to OPMD, documenting the age at their onset as reported by the patient, and a comprehensive neurological examination. Symptoms of ptosis were defined by any perceived upper eyelid drooping by the patient or a history of corrective ptosis surgery. Dysphagia was defined by the patient’s subjective complaints of swallowing difficulties or changes in eating habits. Symptoms related to limb muscle weakness were defined by reported disability, such as difficulty with gait, climbing stairs, standing from a chair, and lifting arms above the shoulders. An evaluation by ENT and ophthalmology specialists was offered to all patients as part of the OPMD clinic services, but was not mandatory.

Upon examination, ptosis was defined by any grade of upper eyelid drooping or previous corrective surgery for ptosis [3]. Dysphagia was evaluated by a neurologist and in most cases also by a multidisciplinary ENT team, including a laryngologist, speech-language pathologist, and nutritionist, to detect any level of dysphagia. The severity of dysphagia was graded according to the Functional Oral Intake Scale (FOIS), which is a 7-level ordinal scale that describes the degree of functional oral intake, ranging from nothing by mouth (Level 1) to total oral diet with no restrictions (Level 7) [28]. Muscle strength was examined bilaterally and scored according to the Medical Research Council (MRC) scale, in which 0 indicates complete paralysis and 5 indicates normal strength. Numerical scoring of intermediate MRC grades was: 5- = 4.75; 4+ = 4.25; 4- = 3.75; 3+ = 3.25; 3- = 2.75. Asymmetry in muscle weakness was defined as a difference of 1 or more in MRC scores between the two body sides.

Patients were encouraged to visit the OPMD clinic annually; however, many did not continue with follow-up. Therefore, reported follow-up details are limited. For this study, we utilized data collected during the initial visit to the OPMD clinic. When available, we collected data on serum creatine kinase (CK) levels, antibodies associated with myasthenia gravis, and pulmonary function tests. Nerve conduction tests (NCT) and electromyography (EMG) were performed at the SMC neuromuscular clinic as previously described [29]. The study was registered at ClinicalTrials.gov (NCT07146256).

Notably, 30 OPMD patients presented in the current study were included in a previous publication about ocular manifestations of OPMD [30], and 8 in a study about the yield of genetic workup and testing in middle-aged and elderly patients with neurological disorders [31].

### Genetic testing

All study participants had a confirmed genetic diagnosis of OPMD. Testing was performed by several certified genetic laboratories in Israel, typically using polymerase chain reaction (PCR) amplification of the specific trinucleotide-repeat region in exon 1 of *PABPN1*, followed by capillary electrophoresis of the products to detect expansions. Some participants completed genetic testing before recruitment to the study, while others underwent testing after their clinic visit, usually at the SMC Genetic Institute.

### Statistical analysis

Patient characteristics were summarized using mean ± standard deviation. To account for family clustering and the repeated-measures structure of the data, linear mixed-effects models (LMMs) were fitted using restricted maximum likelihood (REML) estimation. The first analysis examined overall differences in age of onset across symptom types. This model included random effects for both patient (to account for repeated measures across symptoms) and family (to capture within-family dependency). The second analysis assessed whether the age of onset differed by sex for the various OPMD symptoms. To appropriately model the data structure, LMMs with REML estimation were again used. Separate models were fitted for any symptom and for each specific symptom: dysphagia, ptosis, and muscle weakness, testing the association with sex while including a random effect for family clustering. All analyses were conducted in R software (version 4.5.1) using the *lme4* and *lmerTest* packages. Statistical significance was evaluated at *p* ≤ 0.05.

## Results

### Sample description

The overall sample included 102 symptomatic patients with a confirmed genetic diagnosis of OPMD. Fifty-two of the participants (51.0%) were males, and the mean age at first evaluation (enrolment) in the clinic was 61.4 ± 11.0 (range 36.8 - 82.4) years.

**Fig. 1:**
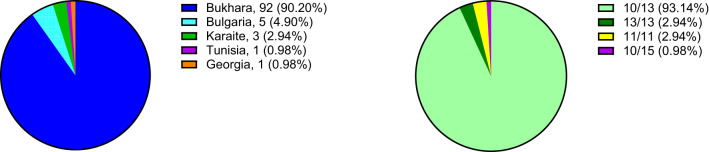
(**A**) The ancestry of all 102 patients with OPMD. (**B**) Distribution of the PABPN1 (GCN)n/n genotype

Patients were from 75 nuclear families, with 46 participants having at least one relative in our smaple, including two participants in 13 families and three or more in six families. All patients were of self-report Jewish origin, and 92/102 (90.2%) were of Bukharian descent (Fig. 1A). Notably, five patients (4.9%) were of Bulgarian descent, and the other five patients were from Georgia (*n* = 1), Tunisia (*n* = 1) and Karaites from Egypt (*n* = 3) (Fig. 1A). In 4/92 (4.3%) of patients of Bukharian descent, only one of the parents was of this origin, but the family history was consistent with disease transmission from the Bukharian parent. Interestingly, only 40/102 (39.2%) participants were born in Israel, and the others immigrated from abroad.

A family history of OPMD diagnosis or typical symptoms in a first, second, or third-degree relative was reported by 92/95 (96.8%) patients, while in seven patients this information was unavailable. Affected parents were reported by 64/92 (67.4%) patients, where, in two cases, both parents were affected. Affected siblings were reported by 49/95 (51.6%) patients, including two or more affected siblings by 21/95 (22.1%), grandparents in 14/95 (14.7%), and affected offspring by 10/95 (10.5%) patients.

### Genetic analysis

The heterozygous (GCN)13 repeat allele in *PABPN1* (10/13 genotype) was identified in 95/102 (93.1%) patients (Fig. 1B), of which 89/95 (93.7%) were of Bukharian, 5/95 (5.3%) Bulgarian, and one (1.1%) of Georgian descent (Table 1).

**Table 1:** Demographic and clinical characteristics of the sample

*PABPN1* Genotype, (GCN)n/n	10/13	10/13	10/13	10/15	13/13	11/11
Origin	Bukhara	Bulgaria	Georgia	Tunisia	Bukhara	Karaite
Participants n (%)	89 (87.3)	5 (4.9)	1 (1.0)	1 (1.0)	3 (2.9)	3 (2.9)
Males n (%)	45 (50.6)	2 (40.0)	0 (0)	0 (0)	2 (66.7)	3 (100)
Age at evaluation						
Mean ± SD	61.3 ± 10.6	65.6 ± 13.6	72.6	61.8	46.5 ± 1.4	76.3 ± 2.6
Range	36.8 - 82.4	43.1 - 76.8	NA	NA	44.9 - 47.4	73.3 - 77.9
First symptom age of onset						
Mean ± SD	50.5 ± 7.7	53 ± 8.5	52	55	39.3 ± 2.5	64.3 ± 4.6
Range	32.0 - 69.0	42.0 - 63.0	NA	NA	37.0 - 42.0	59.0 - 67.0
Dysphagia						
Presence at evaluation n (%)	87 (97.8)	4 (80)	1 (100)	1 (100)	3 (100)	3 (100)
Age of onset: Mean ± SD	52.6 ± 8.2	58.8 ± 6.0	67	55	39.7 ± 3.1	64.3 ± 4.6
Range	32.0 - 73.0	50.0 - 63.0	NA	NA	37.0–43.0	59.0 - 67.0
Ptosis						
Presence at evaluation n (%)	80 (89.9)	4 (80)	1 (100)	1 (100)	3 (100)	2 (66.7)
Age of onset: Mean ± SD	54.0 ± 7.2	53.5 ± 9.3	52	55	40.3 ± 1.5	71.5 ± 2.2
Range	35.0 - 69.0	42.0 - 62.0	NA	NA	39.0 - 42.0	70.0 - 73.0
Muscle weakness						
Presence at evaluation n (%)	51 (57.3)	4 (80)	1 (100)	1 (100)	3 (100)	2 (66.7)
Age of onset: Mean ± SD	57.3 ± 10.1	60.5 ± 13.0	70	60	42.3 ± 2.5	69.5 ± 2.1
Range	35.0 - 76.0	42.0 - 70.0	NA	NA	40.0 - 45.0	68.0 - 71.0

Abbreviation: n. number; NA, not available; SD, standard deviation

A single patient harbored the (GCN)15 repeat allele (10/15 genotype), and was of Tunisian origin. A homozygous expansion was identified in 6/102 (5.9%) patients, with the (GCN)13 repeats (13/13 genotype) in three patients of Bukharian origin, and (GCN)11 repeats (11/11 genotype) in three patients of Karaite ancestry from one family, who immigrated to Israel from Egypt (Fig. 1B and Table 1).

### Clinical characteristics of heterozygous (GCN)13 patients

The onset of symptoms related to OPMD occurred at a mean age of 50.7 ± 7.7 years (*n* = 95). Specifically, the mean onset age was 53.0 ± 8.3 years (*n* = 91) for dysphagia, 53.9 ± 7.2 years (*n* = 80) for ptosis, and 57.9 ± 10.2 years (*n* = 49) for muscle weakness. Dysphagia was the initial symptom in 40/95 (42.1%) patients, ptosis occurred first in 26/95 (27.4%), and limb muscle weakness in 6/95 (6.3%) in our sample. In other cases, initial symptoms appeared in combinations.

**Fig. 2:**
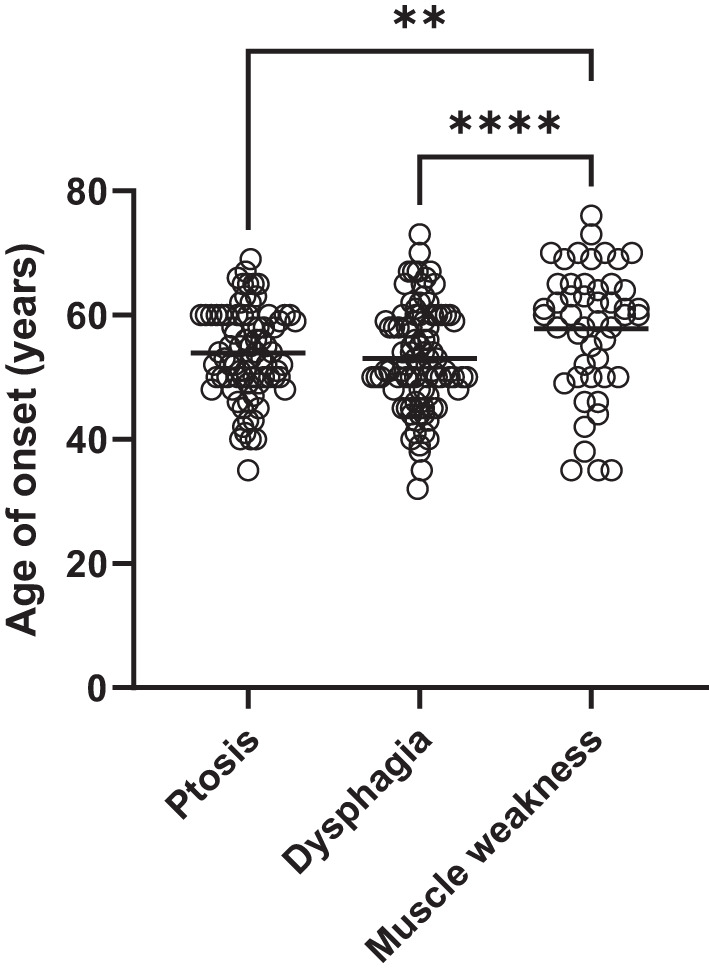
Age of onset for OPMD symptoms among heterozygous (GCN)13 patients (n=95). Specifically, dysphagia was reported by 91 patients, ptosis by 80 and limb muscle weakness by 49. The horizontal line denotes the mean. ** *p* < 0.001; **** *p* < 0.00001 (linear mixed-effects model; Tukey’s adjustment for multiple comparisons)

A mixed-effects model was fitted to examine differences in the age of symptoms’ onset across dysphagia, ptosis, and muscle weakness while accounting for clustering by both patient and family and controlling for sex. The reference symptom was ptosis, with dysphagia occurring at a nonsignificantly earlier age (β = −0.5, *p* = 0.53) and muscle weakness presenting significantly later, on average after 4.4 years (β = 4.4, *p* < 0.001) (Fig. 2).

**Fig. 3:**
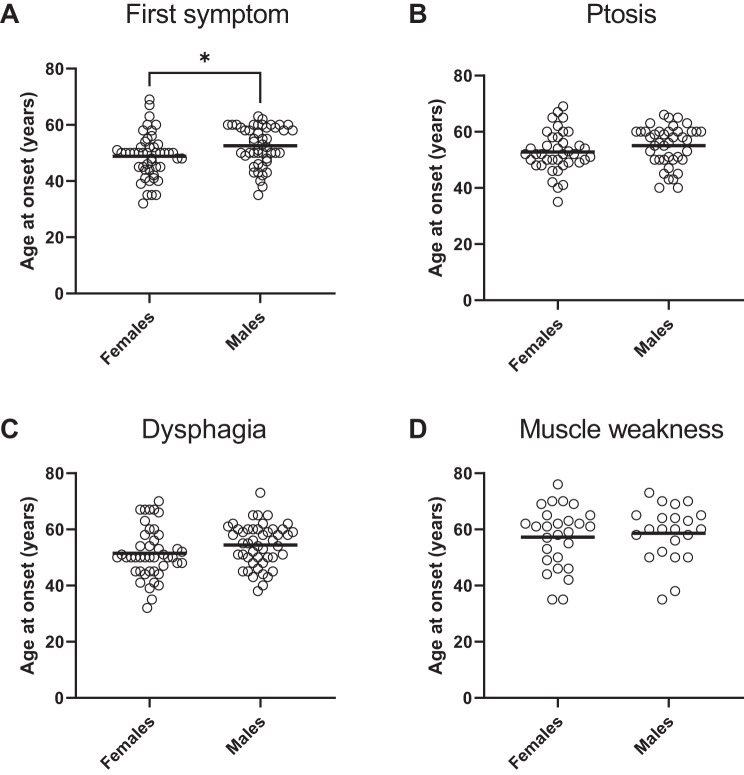
Age of onset for the various OPMD symptoms by sex, among heterozygous (GCN)13 patients (n=95). (**A**) first symptom (n=95), (**B**) ptosis (n=80), (**C**) dysphagia (n=91), and (**D**) limb muscle weakness (n=46). The horizontal line within each graph indicates the mean. * *p* = 0.02 (*t-test* with satterthwaite-adjusted degrees of freedom, and ordinary least squares (OLS) model fitted)

We examined the association between sex and symptom onset, as illustrated in Fig. 3 (A–D). Overall, the onset of symptoms (dysphagia, ptosis, or muscle weakness) occurred approximately 4 years earlier in females (48.9 ± 7.9 years, *n* = 48) compared to males (52.6 ± 7.1 years, *n* = 47), and the difference was significant (*p* = 0.02; Fig. 3A).

Across symptoms, females showed an earlier but nonsignificant mean onset for dysphagia (F: 51.5 ± 8.9 [*n* = 44], M: 54.4 ± 7.5 [*n* = 47]; *p* = 0.10) ptosis, (F: 52.8 ± 7.4 [*n* = 39], M: 55.0 ± 7.0 [*n* = 41]; *p* = 0.10) and muscle weakness (F: 57.2 ± 10.8 [*n* = 27], M: 58.6 ± 9.8 [*n* = 22]; *p* = 0.62). The lack of significance may be attributed to a smaller sample size for each symptom than for the overall onset.

**Fig. 4:**
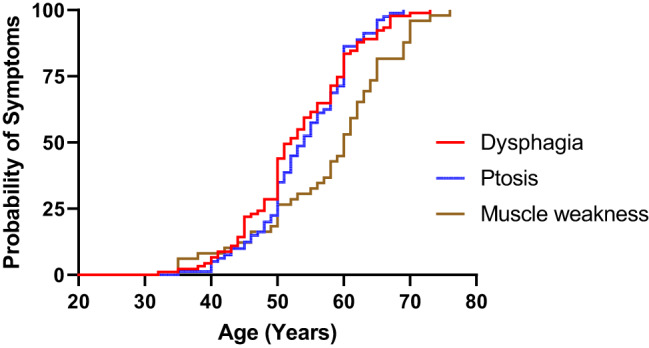
Probability of symptom initiation according to age

The presentation of symptoms increased with age (Fig. 4), and the mean time from symptom onset to confirmed genetic diagnosis was 8.8 ± 7.0 years. The mean interval between symptom onset and assessment at our clinic was 10.9 ± 7.1 years. On initial evaluation, at a mean age of 61.6 ± 10.7 years (*n* = 95, range 36.8–82.4) dysphagia was present in 92/95 (96.8%) of patients, ptosis in 85/95 (89.5%) and muscle weakness in 56/95 (56.9%). Muscle weakness was most notable on testing the lower limbs, with weakness of hip flexion (iliopsoas) detected in 90/188 (47.9%) and hip abduction (gluteus medius) in 72/184 (39.1%) of tested muscles, which was commonly symmetric (Table 2). In the upper limbs, the deltoid muscle most frequently exhibited weakness, whereas the triceps brachii showed slight asymmetry in weakness. Notably, a few patients did not report relevant symptoms but had findings consistent with dysphagia (n=1), ptosis (n=5), and muscle weakness (n=7) in the neurological evaluation.

**Table 2:** Muscle weakness distribution and score in heterozygous (GCN)13 patients

Muscles tested	Tested muscles (N)*	Weak (<MRC 5) muscles (n)	Weak (<MRC 5) muscles (%)	Average MRC score	Asymmetric weakness (%)
Deltoid	188	37	19.7	4.8	9.5
Biceps brachii	188	21	11.2	4.9	23.1
Triceps brachii	188	12	6.4	5.0	28.6
Wrist extensors	120	15	12.5	4.9	20.0
Abductor pollicis brevis	184	13	7.1	4.9	14.3
First dorsal interosseous	182	23	12.6	4.9	15.4
Iliopsoas	188	90	47.9	4.5	6.4
Quadriceps	188	50	26.6	4.8	7.7
Hip Adductors	168	41	24.4	4.7	0.0
Gluteus Medius	184	72	39.1	4.6	5.4
Hamstrings	186	46	24.7	4.8	15.4
Gluteus Maximus	158	27	17.1	4.9	7.1
Tibialis Anterior	186	21	11.3	4.9	8.3

Abbreviation: MRC, Medical Research Council score; n, number

MRC score data were unavailable for one patient

Additional signs and symptoms at evaluation were dysarthria and or dysphonia in 52/95 (54.7%) and diplopia (complaints or on eye movement gaze examination) in 10/95 (10.5%) of patients. Ptosis correction surgery was performed by 55/95 (57.9%) of patients, and in 23/55 (41.8%) cases, a relapse of ptosis occurred. A multidisciplinary ENT team evaluation was performed on 70 patients, and dysphagia was detected in 68/70 (97.1%) of cases. In one of the two patients with no evidence for dysphagia per ENT team exam, symptoms of dysphagia began less than 6 months prior to examination, and in one case, mild swallowing impairment was identified. FOIS was graded as level 4 in one patient (1.4%), level 5 in 11 patients (15.7%), level 6 in 33 (47.1%), and level 7 in 25 (35.7%) patients, indicating food restrictions in 45/70 (64.3%) of patients. Cricopharyngotomy was performed in only 3/95 (3.2%), with only a short relief (<1 year) reported by one. Percutaneous Endoscopic Gastrostomy (PEG) was performed in 2/95 (2.1%). In one case with severe dysphagia, PEG could not be performed due to a history of post-surgical complications. Instead, a distal jujunostomy was performed, followed by ileus and lethal aspiration pneumonia.

A walking aid was required by 8/95 (8.4%) patients at evaluation.

Pulmonary function tests were performed by 28 patients, and showed an average forced vital capacity (FVC) of 80.4% ± 14.2, compared to the expected value for their age. The FVC was below 80% of the expected in 11/28 (39.3%) cases.

EMG was performed in 30 patients and demonstrated myopathy in 16/30 (53.3%) cases, with an indication for fiber disruption per denervation-associated fibrillation potentials in 11/16 (68.8%) cases. Creatinine kinase (CK) measurement was documented at least once in 21 patients, with an average of 274 ± 216 (range 26–800 u/L), and was elevated (>500 u/L) in 3/21 (14.3%) cases. To exclude the diagnosis of myasthenia gravis, eight patients were examined for anti-acetylcholine receptor (AChR) antibodies, which were negative in all cases. In four of the latter, single-fiber SFEMG (SFEMG) was also performed, with negative results.

### Description of additional patients

Three unrelated patients of Bukharian descent, two males and one female, were homozygous for the (GCN)13 allele. Their presenting symptoms occurred on average 11.4 years earlier than those of the hetrozygotes, with dysphagia at the age of 39.7 ± 3.1 years, followed by ptosis and later muscle weakness (Table 1). Three male patients of Karaite origin from a single family were homozygous for the (GCN)11 allele, and their first symptom was dysphagia at age 64.3 ± 4.6 (Table 1). Due to suspected myasthenia gravis, one was tested for anti-AChR antibodies and SFEMG, which were both negative. Last, a single female patient was heterozygous for the (GCN)15 allele. She was of Jewish Tunisian descent, without a known family history of OPMD. Her initial symptoms were dysphagia and ptosis, which appeared together at age 55, followed by limb muscle weakness at age 60 (Table 1). She had weakness of hip flexion and abduction, myopathy with fibrillation potentials on EMG, and normal FVC.

## Discussion

This study summarizes the genetic and clinical data of 102 OPMD patients collected from Israel’s national OPMD registry. Most patients (90.2%) were of Bukharian Jewish ancestry, but few were of other ancestries (Bulgaria, Karaite, and Tunisia). Nearly all patients had a family history of typical symptoms or a diagnosis of OPMD.

Phenotypically, 95 patients who were heterozygous for the (GCN)13 allele had typical symptoms of OPMD, presenting with dysphagia and ptosis, followed later by proximal limb muscle weakness in the sixth decade of life. Overall, the onset of OPMD symptoms was earlier in females compared to males.

Additional symptoms in about half of the patients were dysphonia or dysarthria. The majority of patients required surgical correction for ptosis, while cricopharyngotomy and PEG for dysphagia were uncommonly performed in our patients. The muscle weakness was usually symmetric, with mild asymmetry noted in the triceps brachii muscle in a third of patients. Fewer patients had also distal muscle weakness, e.g., of the tibialis anterior, consistent with a previous report which showed dystrophic changes in this muscle in 17.1% of OPMD patients by muscle MRI scans [32]. We also found mild respiratory impairment in some of the cases, which was previously acknowledged but not commonly reported [33].

In contrast to a previous report published in 1993 regarding the Bukharian Jewish cluster in Israel, which included 78 participants [23], our study indicated that ptosis and dysphagia appear at a similar time. We found that dysphagia was the most common symptom on examination, verified in most cases by an ENT specialist. Notably, all OPMD patients in the current study were genetically confirmed, allowing us to selectively focus on characteristics of patients with the heterozygous (GCN)13 allele.

In addition, our study included five patients of Bulgarian descent. A cluster of OPMD patients from this origin was previously described, with a presentation including early shoulder girdle weakness in addition to oculopharyngeal features [34]. In our evaluation of five patients of Bulgarian origin, the initial symptom was ptosis, and limb weakness was the last to appear.

A similar age of onset for both ptosis and dysphagia in OPMD patients with the (GCN)10/13 genotype was previously reported in the French-Canadian population [6], where the median age of onset for ptosis and dysphagia was 54 years, comparable to our sample. Lower-limb proximal weakness was reported, with a median age of onset of 58 years, which is similar to our sample. Richards and collaborators have suggested a correlation between the length of the (GCN) repeat and the severity of OPMD [2]. They showed that patients with a longer repeat expansion in *PABNP*1 were diagnosed at an earlier age, compared to those with a shorter expansion. In France, the mean age at diagnosis of OPMD patients was 64 ± 10 years for (GCN)10/13 genotype patients, compared to 60 ± 10 for (GCN)10/15 [2]. Among 25 patients with the (GCN)10/13 genotype from Spain [35], the mean age of symptom onset was 54 ± 5 years, while in 113 OPMD patients from the Canary Islands, all heterozygous for the (GCN)15 allele, the mean age of onset was 45 ± 6.4 years [26].

However, a direct comparison of the age of onset between various populations is challenging due to multiple factors, among them the mix of genotypes, differences in study design, data collection methods, and potential environmental exposures.

Our finding that the onset of symptoms in females was earlier than in men is intriguing and has not been previously reported. Generally, sex effect on age of onset in myopathies is related to multiple mechanisms, such as baseline muscle mass, the effect of estrogen and testosterone and epigenetic regulation [36–38]. In OPMD, age at onset is attributed to the repeat expansion length (as mentioned above), and further studies are required to validate our finding of earlier symptoms onset in females among patients with 10/13 and possibly other genotypes.

In our study, three homozygous patients for the (GCN)13 repeat exhibited a similar pattern of symptoms, albeit with an onset 11.4 years earlier, indicating a severe phenotype consistent with a biallelic gene-dosage effect. In this small group, dysphagia was the common initial symptom, similar to that in the heterozygote patient cohort. In contrast, three patients, homozygous for the (GCN)11 repeats of Karaite origin, had initial symptoms only in their 60s and 70s. Indeed, the homozygous (GCN)11 allele causes OPMD with mild manifestations. Interestingly, the (GCN)11 allele, which has a frequency of 1–2% in some populations, may act as a disease modifier of OPMD caused by a larger repeat expansion [3, 11, 39].

The findings presented in this study are based on the IsrO-PMD registry database, which aims to describe the natural history of OPMD through a multidisciplinary approach and establish a platform to facilitate and advance clinical and translational research of OPMD. The methodology and protocol of the database were previously described [27]. Recently, a detailed description of the ophthalmological manifestations in 30 OPMD patients from our multidisciplinary clinic was published, raising awareness of ocular features other than ptosis, including abnormalities in eye movements, strabismus, and potential diplopia [30]. The majority of our patients required surgical correction for ptosis. However, in approximately one-quarter of cases, the procedure did not result in a lasting improvement. Nevertheless, ptosis is surgically correctable with a relatively brief procedure, typically performed under local anesthesia and lasting about one hour. Given the significant impact of ptosis on patients’ quality of life and daily functioning, surgical intervention should be considered for affected patients. On the contrary, we observed a low rate of cricopharyngeal myotomy, particularly given the high prevalence of dysphagia in our patients. Although the explanation for this is unclear, we believe it originates from recognition of the recurrence rate even at specialized centers, which was reported to be about a third over three years [40].

While OPMD has a unique phenotype and is relatively prevalent in Israel, awareness among medical care providers to this condition is still limited. For example, nine patients underwent testing for myasthenia gravis, which may resemble OPMD due to the presence of ptosis, dysphagia, and limb weakness, but differs in manifestations of fatigue, asymmetry of ptosis, presence of activity-induced diplopia, and absence of a family history of similar symptoms. Accordingly, all these patients were found to be negative for anti-AChR antibodies, although these conditions may rarely coexist [41].

Several limitations of the current study should be acknowledged. The sample consists of 102 patients (92 of Bukharian descent), while we expect the number of OPMD patients living in Israel to be substantially larger. Therefore, the sample underrepresents the total Israeli OPMD population. A prior report in 1997 estimated OPMD minimal prevalence at 1:600 among 70,000 Bukhara Jews living in Israel [20]. Official data on the current size of this population in Israel are unavailable; however, assuming the population size is now at least twice as large, the expected number of OPMD patients may be estimated at approximately 233. Indeed, we are aware that a few OPMD patients with a molecular diagnosis or a high level of clinical suspicion declined joining the registry and completing evaluation for various reasons. Some were elderly and more physically disabled, and consequently had difficulty accessing the national clinic, while others were uninterested in the study. As the IsrO-PMD registry expands in the coming years and awareness of this initiative increases among patients and medical service providers, the sample size will likely increase. In addition, the data on age at onset and initial symptoms were retrospectively collected and may, therefore, be influenced by recall bias and subjectivity of self-reported symptoms, rendering them less accurate.

We report data from the baseline visit in the neuromuscular clinic, but we expect that most participants will continue to be followed, allowing collection of longitudinal data in order to better characterize disease progression and natural history at later stages.

Lastly, other important aspects of deep OPMD phenotyping were not routinely employed in our patients, such as quantitative muscle strength by dynamometry, video fluoroscopy of swallowing, magnetic resonance imaging (MRI) and ultrasound imaging of the muscles, and cardiac assessment. For example, muscle MRI of the tongue, adductor magnus, and soleus commonly shows impairment and correlates with disease duration [32]. Additional features of OPMD manifestations should be investigated in more detail, in particular, swallowing difficulties, cognitive impairment, fatigue, emotional stress, and long-term outcomes of surgical interventions. For instance, it has been suggested that executive functions are impaired in OPMD patients [42]. Several of these are currently collected at our clinic to obtain a multi-dimensional assessment for future exploration.

From the therapeutic perspective, several treatment options for OPMD have been investigated in recent years, including anti-aggregators, myostatin inhibitors, autologous myoblast transplantation, and gene therapy [43–49]. As clinical trials for OPMD emerge, there is a need to assess clinical outcomes and potential biomarkers more effectively to determine the rate of disease progression, predict prognosis, and monitor future treatments [4, 50]. In addition to testing functional capacities and muscle imaging (ultrasonography and MRI), other biomarkers for OPMD are based on metabolomic, proteomic and miRNA analysis in blood or saliva [50–53]. These aspects should be integrated into a longitudinal study of the Israeli OPMD registry, in order to validate findings reported elsewhere and to discover novel ones.

## Conclusions

This study comprehensively describes the clinical features of OPMD patients in Israel, primarily among a relatively large group of patients with the (GCN)10/13 genotype. Since OPMD is a rare disease, our study provides valuable data about demographic and clinical characteristics that are comparable to observations reported in samples of OPMD patients from other populations.

## Data Availability

The datasets used and analysed during the current study are available from the corresponding author on reasonable request.
